# INFLAMMATORY CYTOKINES MEDIATE EXECUTIVE FUNCTION AND STRESS AMONG AGING ADULTS

**DOI:** 10.1093/geroni/igad104.3432

**Published:** 2023-12-21

**Authors:** Julie Kircher, Susan Charles, David Almeida

**Affiliations:** University of California, Irvine, Irvine, California, United States; University of California, Irvine, Irvine, California, United States; The Pennsylvania State University, State College, Pennsylvania, United States

## Abstract

Strong and consistent evidence demonstrates that a risk factor for poorer executive function is elevated levels of perceived stress. More recently, researchers have posited that inflammatory processes may contribute to the association between stress and cognitive declines. Similarly, research suggests that greater sustained inflammation can result from higher levels of stress. Furthermore, older adults are at a higher risk of experiencing cognitive decline when under greater stress. We hypothesized that inflammation, as measured by a composite of pro-inflammatory cytokines CRP, IL-6, and TNF-a, would mediate the relationship between perceived stress and executive function. Data were derived from the cognitive and biomarker projects in the second and third waves of the Midlife in the United States Study (MIDUS). The present study included 1,067 participants (Mage = 63.7; Range 39 - 94). The results showed a significant total effect between executive function and perceived stress (β = .05, p <.001). Furthermore, path analyses revealed significant pathways between stress on inflammation (β = 0.29 p <.001) and inflammation on executive function (β = .06, p <.001). Finally, when inflammation was entered into the relationship between perceived stress and executive function its direct effect was significant (β = 0.06, p <.001). These findings suggest that pro-inflammatory cytokines help to explain why perceived stress is related to lower capability in executive functions. Future research should focus on understanding and assessing the interactions between inflammation, stress, and cognitive functioning over time.

